# CRISPRi screen uncovers lncRNA regulators of human monocyte growth

**DOI:** 10.1016/j.jbc.2025.110204

**Published:** 2025-05-07

**Authors:** Cristina Flores-Arenas, Eric Malekos, Christy Montano, Sergio Covarrubias, Lisa Sudek, Valory Dempsey, Vuky Huynh, Sol Katzman, Susan Carpenter

**Affiliations:** 1 Department of Molecular, Cell and Developmental Biology, University of California Santa Cruz, California, USA; 2 Department of Biomolecular Engineering, University of California Santa Cruz, California, USA

**Keywords:** Long noncoding RNAs, monocytes, CRISPR, high throughput pooled screen, *LNCRMP*, *OLMALINC*, CRISPR inhibition, cis regulation

## Abstract

Long noncoding RNAs are emerging as critical regulators of biological processes. While there are over 36,000 lncRNAs annotated in the human genome, we do not know the function of the majority. Here, we performed a high-throughput CRISPRi screen to identify those lncRNAs that are important for viability in human monocytes using the cell line THP1. We identified a total of 38 hits from the screen and validated and characterized two of the top intergenic hits. The first is a lncRNA neighboring the macrophage viability transcription factor IRF8 (*RP11-542M13.2* hereafter referred to as long noncoding RNA regulator of monocyte proliferation, *LNCRMP*), and the second is a lncRNA called *OLMALINC* (oligodendrocyte maturation-associated long intervening non-coding RNA) that was previously found to be important in oligodendrocyte maturation. Transcriptional repression of *LNCRMP and OLMALINC* from monocytes severely limited their proliferation capabilities. RNA-seq analysis of knockdown lines showed that *LNCRMP* regulated proapoptotic pathways, while knockdown of *OLMALINC* impacted genes associated with the cell cycle. Data support both *LNCRMP* and *OLMALINC* functioning *in cis* to regulate their neighboring proteins that are also essential for THP1 cell growth. This research highlights the importance of high-throughput screening as a powerful tool for quickly discovering functional long non-coding RNAs (lncRNAs) that play a vital role in regulating monocyte viability.

Monocytes are the largest white blood cells circulating in the periphery that play critical roles in regulating innate immune responses (1). Key features of monocytes are their ability to sense pathogen-associated molecular patterns, proliferate, and migrate to tissues where they differentiate into macrophages or dendritic cells (2, 3). Both monocytes and macrophages are critical to the activation and resolution of inflammation through phagocytosis, initiation of cytokine and chemokine signaling cascades, and activation of the adaptive immune system (3, 4). Under normal homeostatic conditions, circulating monocytes are short-lived and die by apoptosis (4). Increased accumulation of monocytes can promote diseases including cancer, cardiovascular disease, liver fibrosis, and multiple sclerosis (5). Considering the vast number of biological processes monocytes take part in, including development, wound healing, and cancer progression (4, 6), we sought to determine what role long noncoding RNAs might play in monocyte growth and proliferation.

Long noncoding RNAs represent the largest group of RNA produced from the human genome. LncRNAs are defined as transcripts longer than 500 nucleotides, often spliced and polyadenylated, but without the ability to code for proteins (7). In recent years, research has revealed diverse roles for lncRNAs in various biological processes, such as cell differentiation, cancer, and immunity (8, 9, 10, 11). However, the functions of the vast majority of lncRNAs remain unknown. A significant hurdle in studying lncRNAs has been the lack of reliable and specific methods for targeting them, especially in high-throughput settings. Traditional CRISPR/Cas9 techniques, which rely on inducing frameshift mutations, are ineffective against lncRNAs as they lack open reading frames. The emergence of CRISPR inhibition (CRISPRi), a modified CRISPR/Cas9 technology, has provided a powerful solution. CRISPRi can silence lncRNA transcription by inducing repressive chromatin marks at their start sites, making it a valuable tool for uncovering lncRNA functions. While lncRNA expression profiles have been examined in human monocytes (12) there is a lack of functional data and a small number of lncRNAs have been identified as playing roles in monocyte differentiation, there is a lack of systematic approaches to identify those lncRNAs important in monocyte survival (13, 14).

Liu, Horlbeck *et al.* published one of the first lncRNA screens aimed at understanding which lncRNAs are essential in human cells (15). They profiled seven cell lines identifying a total of 499 lncRNA using high throughput pooled CRISPRi screening. Interestingly, the majority of the hits were unique to each cell type, indicating that lncRNAs are highly cell and tissue-specific in expression and function. We recently utilized a similar approach to identify all lncRNAs involved in monocyte-induced inflammation and monocyte to macrophage differentiation (9, 16). Here we applied a similar approach, utilizing our THP1-specific lncRNA library (16) to profile and determine which lncRNAs are important to monocyte survival and proliferation. Of the top 35 hits involved in cell monocyte cell viability, 18 are antisense transcripts, eight are intergenic, four are now annotated as protein coding, three are intronic, and two are bidirectional transcripts. We performed mechanistic studies on two hits, *LNCRMP* and *OLMALINC.* RNA-sequencing following knockdown of each lncRNA revealed that they both function to regulate viability in different ways. While both genes can impact their neighbor *in cis OLMALINC* regulates cell cycle pathways, while *LNCRMP* impacts interferon signaling as well as pro-apoptotic proteins. Here, we present the power of CRISPR screening to identify functional lncRNAs important in cell growth in human monocytic cells.

## Results

### High-throughput CRISPR screening identifies lncRNAs involved in THP1 monocyte cell growth

**Figure 1:**
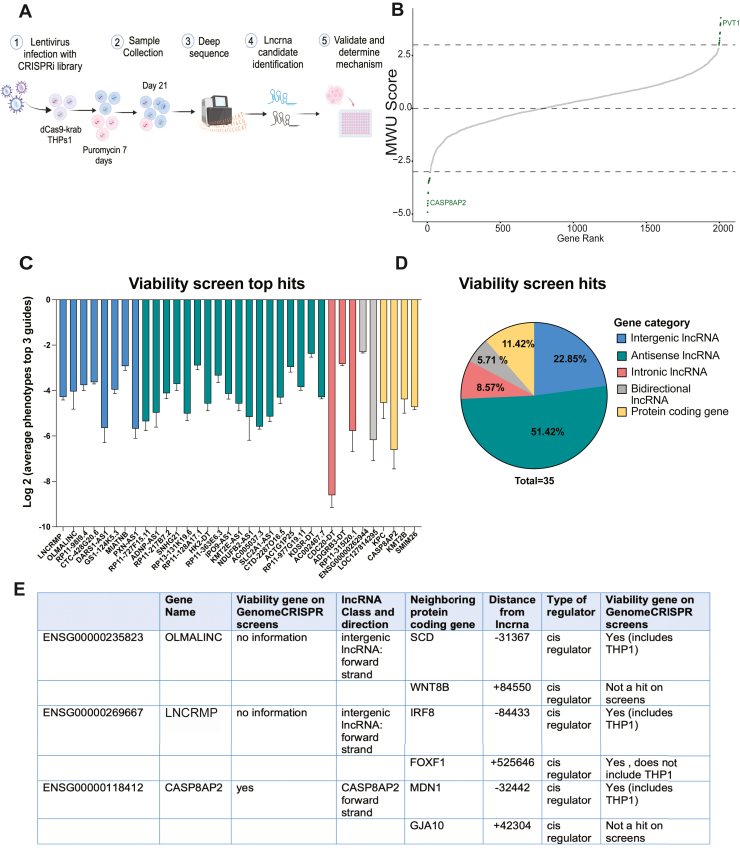
**CRISPRi screen identifies lncRNAs that impact macrophage cell growth.***A*, schematic of drop out CRISPRi screen pipeline. sgRNAs were designed to target the transcription start sites of over 2000 Gencode hg19 annotated lncRNAs. Transcription start sites were predicted using data from FANTOM and ENCODE. The THP1 lncRNA expression was estimated from THP1 RNAseq data. NFkB-EGFP-CRISPRi-THP1 cells were infected with pooled sgRNA libraries. The cells were selected with puromycin, and samples were collected at day 21 to identify the sgrnas that dropped out from the original library. sgRNAs from the library and day 21 were PCR amplified and sequenced. *B*, screen hits. Mann Whitney U analysis was used to determine the gene ranking from the RNA seq data. *C*, top-performing sgRNAs. Top three guides for each significant hit are represented and color coded to match their gene category in (*D*). *D*, ncRNA hit categories. Pie chart for the different categories of lncrnas in the CRISPRi screen. Intergenic lncrnas are lncrnas localized between two genes and they have their own transcription start site (TSS). Antisense lncrnas are lncrnas that transcribed from the same gene locus in the opposite direction. Intronic lncrnas are localized within an intron of a gene. Bidirectional lncRNAs are 1kb away from the gene locus and they are transcribed in the opposite direction. *E*, table summarizing top candidate lncRNAs, *OLMALINC* and *LNCRMP* and a positive control hit *CASP8AP2*.

To identify lncRNAs important in the regulation of monocytic cell growth, we took our recently generated monocytic specific lncRNA CRISPR library and infected THP1 cells (16). The library contains ∼25,000 individual single guide RNAs (sgRNAs) with 10 sgRNAs targeting the transcription start sites of 2342 lncRNAs annotated in the human genome assembly GRCh37 (hg19). The same design and cloning strategies were used as previously described (15) and is presented in Figure 1*A*. THP1s were infected and selected with puromycin for 7 days. After 21 days, genomic DNA was extracted and compared to the starting library to identify the sgRNAs that dropped out or enriched over time. Hits were ranked by comparing fold-change of TSS-targeting sgRNAs to non-targeting control sgRNAs and assigning each a z-score (Fig. 1*B*). Genes with a score of less than −3 were defined as significant positive regulators of THP1 growth while genes above three were defined as significant negative regulators of growth (Fig. 1*B*). Highlighted in the figure are positive control gene hits *PVT1* and *CASP8AP2*. *PVT1* was previously shown to act as a growth suppressor and *CASP8AP2* is a viability gene (17, 18, 19). Here we are only focusing on the sgRNAs that dropped out representing positive regulators of growth in THP1 cells. In Figure 1*C*, we rank the top 35 drop out hits based on the three performing sgRNAs (Fig. 1*C*, Table S1). 18 lncRNA hits are antisense to a protein coding gene, eight hits are intergenic, defined as having their own promoters at least 1kb away from promoters of neighboring genes, four hits are protein coding (previously annotated as noncoding in older genome build), three are intronic and two are from a bidirectional promoter (Fig. 1*D*, Table S2). One of the top hits was CASP8AP2 which was previously identified in a CRISPRi screen as an essential viability factor in lung cancer (Fig. 1*E*) (18). For the remainder of this study, we focus on two hits which were selected as both intergenic long noncoding RNAs that have not been implicated in monocyte or macrophage biology previously (Fig. 1*E*). *OLMALINC* neighbors the protein coding gene stearoyl-CoA desaturase (SCD) and has previously been shown to regulate SCD *in cis* in neuronal cells (20). Interestingly SCD itself has previously been reported to be a viability gene in THP1s in the genome CRISPR screens database (Fig. 1*E*) as well as in the Broads depmap portal https://depmap.org/portal/cell_line/ACH-000146 (gene effect in THP1s of −1.081222 and a z score of −0.63). The second lncRNA RP11-542M13.2 (AC092723.1), which we call lncRNA regulating monocyte proliferation—*LNCRMP*—was selected as it neighbors the important protein coding gene IRF8. We previously reported that IRF8 is a macrophage viability gene in mouse bone marrow derived macrophages, and indeed it has also been reported to act as a viability gene in THP1 screens (21) and Figure 1*E* and in the depmap portal with a gene effect in THP1s of −1.18 and a Z score of −7.85.

### Validation of the top hits *OLMALINC* and *LNCRMP* as regulators of THP1 monocyte growth

**Figure 2:**
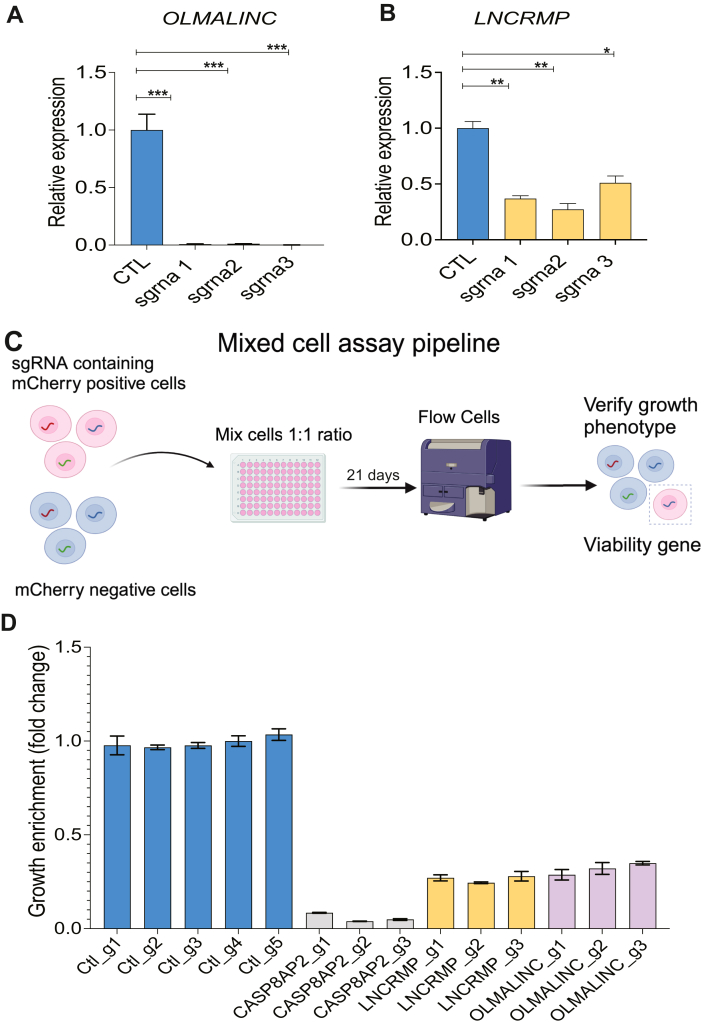
**Validation of *OLMALINC* and *LNCRMP* as hits involved in macrophage growth CRISPRi knockdown of *OLMALINC* and *LNCRMP*.***A* and *B*, sgRNAs (sgRNAs 1, 2, 3) were designed to target top lncRNA candidates *OLMALINC* and *LNCRMP*. qPCR measurement of both genes across three replicate experiments shows knockdown of *OLMALINC (A) and LNCRMP (B)* by all three sgRNAs (∗*p*-values<0.05, ∗∗*p*-values<0.01 and ∗∗∗*p*-values<0.005) vs a non-targeting control sgRNA. *C*, schematic of macrophage growth validation assay. Mcherry positive cells sgRNA containing cells were mixed with mcherry negative cells in a 1:1 ratio and plated in triplicate for each sgrna for each gene. The cells were flowed every other day for 21 days. Mcherry enrichment was then calculated to determine the cell growth. *D*, mixed cell assay results for *OLMALINC* and *LNCRMP*. We combined cherry-positive cells (containing sgRNAs) with unedited cherry-negative cells at a 1:1 ratio and monitored the growth of sgRNA-infected cells (cherry) relative to uninfected reference cells in a mixed-cell growth assay for 21 days. The experiment was repeated 3 times, and a representative experiment was displayed.

We sought to better characterize *OLMALINC* and *LNCRMP*, our candidate hits. To this end, we generated three independent sgRNA lines targeting each gene and confirmed knockdown by qPCR compared to a scrambled control line (Fig. 2, *A* and *B*), consistently achieving >95% knockdown of *OLMALINC*, and 50 to 60% for *LNCRMP*. Due to these genes being associated with cell growth and viability, all phenotypes were assessed after 10 days of puromycin selection to minimize the selection of cells without knockdown. After validating CRISPRi knockdown, we performed a mixed cell assay to validate these genes as regulators of cell growth (Fig. 2*C*). We combined cherry-positive cells (containing sgRNAs) with cherry-negative cells at a 1:1 ratio and monitored cell growth over time as assessed by changes in the ratio of cherry-positive to cherry-negative cells as measured by flow cytometry. All three sgRNAs targeting either *OLMALINC*, *LNCRMP,* or our positive control *CASP8AP2* resulted in between 75% and 95% decreased cell growth compared to the five negative control sgRNAs (Fig. 2*D*). This sensitive assay confirmed that both *OLMALINC* and *LNCRMP* contribute to THP1 cell growth.

### *OLMALINC* is a cytosolic gene regulating monocyte growth *via* SCD and immune/cell cycle checkpoints

**Figure 3:**
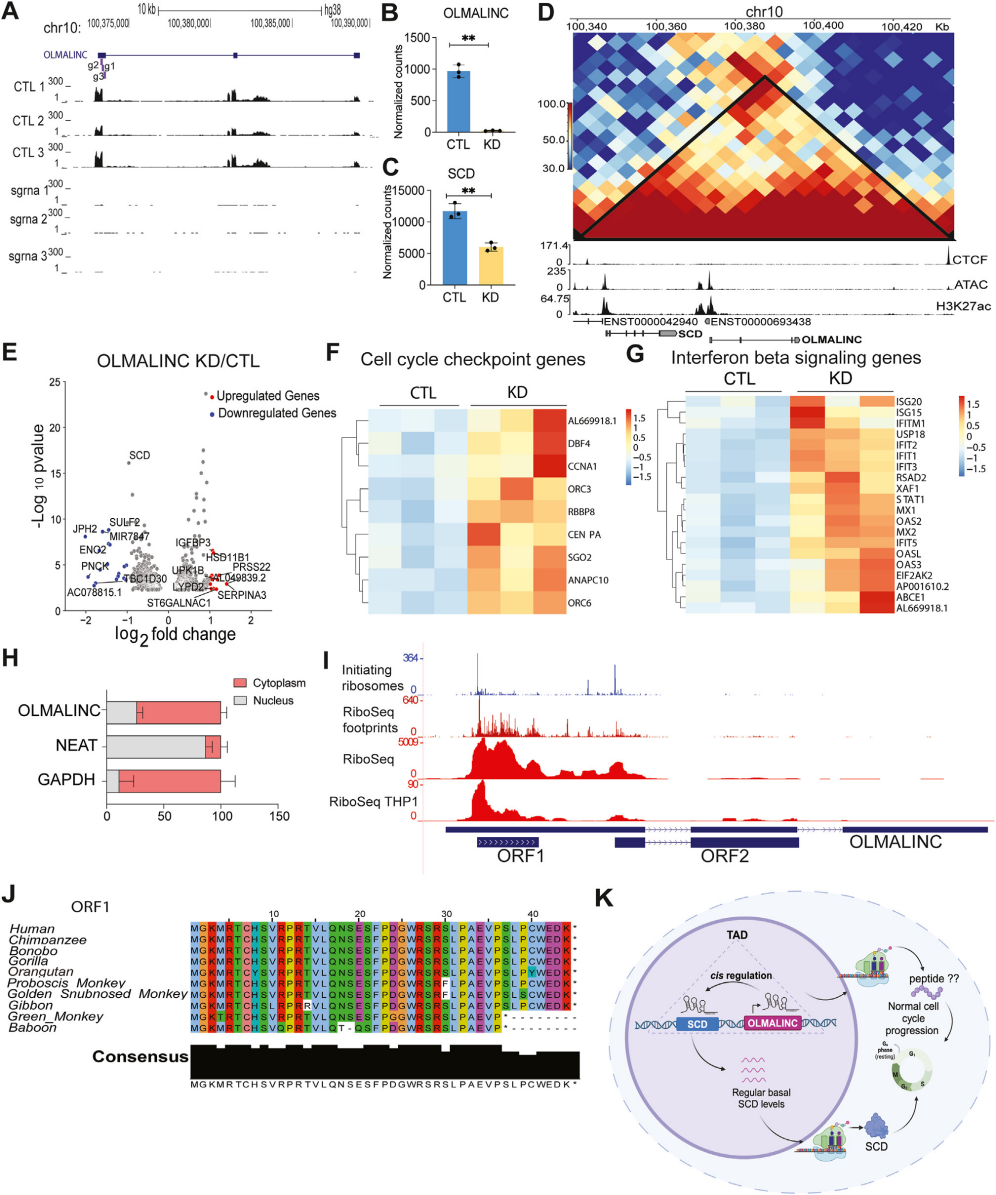
**Functional characterization of *OLMALINC’s* regulation of monocyte growth.***A–C*, CRISPRi knockdown of *OLMALINC* impacts SCD expression. *A*, UCSC genome browser track displays RNA-Seq reads from three control (Ctl) and 3 *OLMALINC* knockdown (KD) cell lines at the *OLMALINC* genomic locus. *B–C*, Normalized read counts from DEseq analysis for *OLMALINC* (*B*) and its neighboring protein SCD (*C*) error bars represent standard deviations of the three biological replicates, *∗∗p < 0.01*. *D*, *OLMALINC* and *SCD* share a TAD. *OLMALINC* and SCD are part of the same topologically associated domain (TAD). Indicating a close genomic proximity and cis-regulation (data analyzed from GSE60857). *E*, differentially expressed genes following *OLMALINC* knockdown. Volcano plot displaying significantly upregulated and downregulated genes in *OLMALINC* KD cells. *F–G*, heatmaps of top 20 upregulated or downregulated genes in *OMALINC* KD cells and control cells. *H*, *OLMALINC* is expressed in the cytosol. RT-qPCR analysis of *OLMALINC* expression in cytoplasmic and nuclear fractions from THP1 cells. The experiment was performed in triplicate, and error bars represent standard deviations of the replicates. *I*, *OLMALINC* possibly encodes short peptides. Riboseq data for *OLMALINC* shows Riboseq aggregates at open reading frame 1 (ORF1) and conservation between different species. Track one shows initiating Riboseq aggregates in the different ORFs. Track two shows Riboseq footprint aggregates. Track three shows Riboseq coverage aggregate. Track four shows Riboseq coverage in THP1 cells. *J*, *OLMALINC* Peptide conservation. Consensus conserved sequence is observed in different species such as human, gorilla, and green monkey. *K*, proposed working model Briefly, *OLMALINC* acts as a *cis-regulator* of SCD (viability coding gene) and regulates its expression. Under basal conditions, *OLMALINC* is expressed at basal levels, and regular levels of SCD are expressed, which results in a normal cell cycle. In the absence of *OLMALINC*, SCD levels are lower, and cell cycle is altered impacting cell growth.

*OLMALINC* has previously been reported to act *in cis* regulating its neighboring coding gene, SCD (20, 22). To determine if there could be a similar mechanism at play in our THP1 cell system, we first performed RNA-sequencing comparing negative control sgRNAs to three sgRNAs targeting *OLMALINC* outlined in the UCSC browser track in Figure 3*A*. We saw a robust knockdown of *OLMALINC*, consistent with our qPCR results (Fig. 3, *A* and *B*). Expression levels of *OLMALINC*’s neighboring protein coding gene, *SCD*, was also significantly reduced, consistent with *cis* regulatory activity. *Cis* acting elements are known to frequently occupy the same TADs as their target; therefore, we looked at THP1 HiC data (analyzed from GSE60857) (23) and confirmed that SCD and *OLMALINC* share a TAD as determined by HiC interaction data and consistent with TAD boundaries determined by CTCF peaks (Fig. 3*D*). This is consistent with a previous study indicating that *OLMALINC* works *in cis* through an enhancer RNA mechanism (22). Knockdown of *OLMALINC* resulted in dysregulation of a number of protein coding genes (Fig. 3*E*), including proteins such as DBF4-CDC7 kinase regulatory subunit A (DBF4) and CCN family member 4,CCNA4 involved in cell cycle control (Fig. 3*F*). In addition to 2′,5′-oligoadenylate synthetase (OAS) proteins and interferon-induced proteins with tetratricopeptide repeats (IFITs) (Fig. 3*G*), including IFIT2 (Fig. S1) involved in Interferon signaling (Fig. 3*G*).

To gain further functional insight into *OLMALINC*, we first determined its localization by fractionation of THP1 cells followed by qRT-PCR. NEAT1 was measured as a nuclear control and GAPDH served as the cytosolic control (Fig. 3*H*). *OLMALINC* is dominantly expressed in the cytosol with some expression (∼30%) in the nuclear compartment (Fig. 3*H*). Considering the high levels of *OLMALINC* localized in the cytosol we next asked if the locus is indeed noncoding or possibly encoding a short, encoded peptide. Previous work by Presener *et al.* identified *OLMALINC* as a gene important in viability in cancer cells. They also showed that it harbored an open reading frame (ORF) and active Cas9 targeting the coding region affected viability in HELA cells (24). Indeed Riboseq, including initiating ribosome reads, Riboseq footprints aggregated by GWIPS, and Riboseq from THP1 cells indicated that there are two translated ORFs within the *OLMALINC* locus. In THP1s, the strongest Riboseq signals support open reading frame 1 (ORF1) and we find that this peptide sequence is highly conserved across primates (Fig. 3*J*). This data suggests that there could be dual roles for this gene locus. Nuclear *OLMALINC* could function *in cis* to regulate its neighboring protein coding gene. It is also possible that the functional peptides contribute THP1 cell growth either through regulating SCD or other gene networks (Fig. 3*K*).

### *LNCRMP* is expressed in both the cytosol and nucleus and regulates both its neighboring gene *IRF8* and other IFN genes

**Figure 4:**
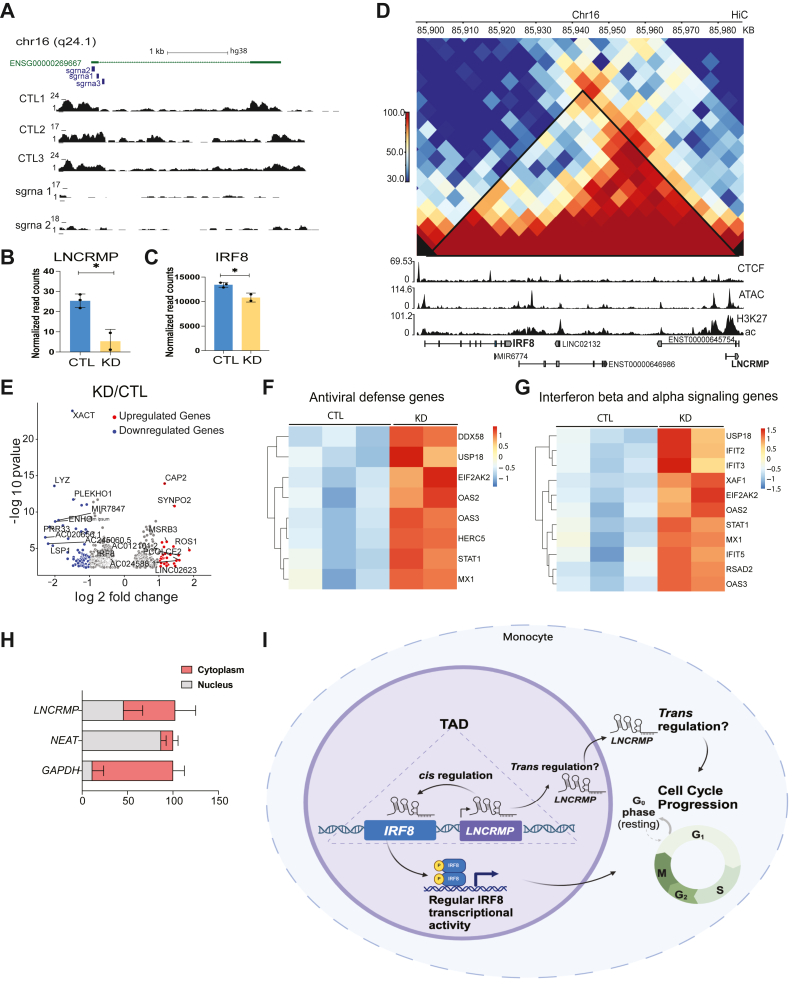
**Characterization of *LNCRMP*’s role in monocytic growth and function.***A*, UCSC genome browser track displays RNA-Seq reads from three control (Ctl) non-targeting sgRNAs and 2 *LNCRMP* knockdown (KD) cell lines at the *LNCRMP* genomic locus. *B*–*C*, expression of *LNCRMP* (*B*) and its neighboring protein SCD (*C*) from RNA-seq data comparing 3 Ctl sgRNAS to 2 *LNCRMP* sgRNA. Error bars represent standard deviations of the biological replicates, *∗p < 0.05*. *D*, *LNCRMP* and IRF8 are part of the same topological associated domain (TAD). Indicating a close genomic proximity and possible cis regulation (data analyzed from GSE60857. *E*, volcano plot displaying significantly upregulated and downregulated genes in *LNCRMP* KD cells. *F–G*, heatmaps of top upregulated *LNCRMP* KD cells and controls across two categories outlined. *H*, RT-qPCR analysis of *LNCRMP* expression in cytoplasmic and nuclear fractions from THP1 cells. The experiment was performed in triplicate and error bars represent standard deviations of the replicates. *I*, proposed working model of how *LNCRMP* acts as a regulator of THP1 viability. Briefly, *LNCRMP* acts in *cis* to regulate IRF8.

To better understand *LNCRMP*’s role in regulating monocyte growth we performed RNA-seq to determine what genes are altered when *LNCRMP*s expression is reduced. UCSC Browser tracks of the *LNCRMP* locus confirmed successful knockdown in the two sgRNAs utilized compared to negative controls (Fig. 4*A*). Normalized read counts from RNA-seq indicate successful knockdown of *LNCRMP* which resulted in small but significant downregulation of its neighboring gene *IRF8* (Fig. 4, *B* and *C*). Utilizing HiC data, we found that both *LNCRMP* and *IRF8* occupy the same TAD within THP1s and is suggestive of a *cis* regulatory effect between the two genes (Fig. 4*D*). Figure 4*E* shows a volcano plot of dysregulated genes following *LNCRMP* knockdown. Significantly upregulated genes with a log_2_ fold change of one are in red and downregulated genes with a log_2_ fold change of −1 are in blue. The top 20 upregulated genes in the *LNCRMP* deficient cells fall into the defense and IFN signaling GO terms indicative of a role for *LNCRMP* in regulating this pathway (Fig. 4, *F* and *G*). Increased baseline expression of IFN inducible genes MX1 and IFIT2 were confirmed by qPCR in LNCRMP knockdown cells (Fig. S2). Using fractionation followed by qRT-PCR we found that *LNCRMP* is expressed in both nuclear and cytosolic compartments (Fig. 4*H*). Given *LNCRMPs* expression in the cytosol we examined THP1 Riboseq data but did not identify any significant reads in the region suggestive of *LNCRMP* being a bonafide noncoding RNA (Fig. S3). Previously, we identified IRF8 as a viability gene in mouse macrophages and our proposed model based on the current findings is that *LNCRMP* can function *in cis* to regulate IRF8 which could have a downstream impact resulting in monocytic cell death (Fig. 4*I*). This is consistent with previous reports on this lncRNA that showed it can function to regulate its neighbor IRF8 through an enhancer RNA mechanism involving recruitment of TET1 to the IRF8 promoter regulating IRF8 expression by affecting methylation levels in the U937 monocytic cell line (25).

## Discussion

Here we present a CRISPRi-based screen in the human monocytic cell line THP1 identifying lncRNAs that regulate cell growth. We focused on two top hits: *LNCRMP* and *OLMALINC*. Interestingly, neither lncRNA was identified in a previous screen of 499 lncRNAs involved in cell growth across seven different cell lines (15). This discrepancy likely reflects cell-type-specific roles for these lncRNAs. *LNCRMP* was not expressed in any of the cell lines tested by Liu *et al.*, while *OLMALINC* was expressed at varying levels (TPMs 0.7–29) but did not significantly affect growth in those contexts. These findings underscore the importance of cell type context in lncRNA function, consistent with Liu *et al.*'s observation that cell type-specific expression was a major determinant of growth regulation. For example, they found that LINC00263, despite widespread expression, was only required for growth in U87 cells (15). It is also possible that other cell types have built-in redundancy and therefore are not reliant on a particular lncRNA.

We have previously reported that protein coding genes can play specific roles in controlling growth in macrophages (21). We performed a whole genome wide active CRISPR screen and identified a total of 61 macrophage specific protein hits (21). Interestingly, one of the hits is the transcription factor IRF8. IRF8 is a key transcription factor that plays roles in macrophage development, can act as a tumor suppressor, and function to regulate inflammatory signaling (26, 27, 28, 29). The locus surrounding human IRF8 is highly complex containing multiple possible lncRNA genes according to Gencode annotations. Despite this, we only identified the region encoding *LNCRMP* as a hit in THP1s. It is possible that the other lncRNAs play functional roles in other cell types. Interestingly, a previous report by Zhou *et al.* identified a Lupus disease associated variant rs2280381 which lies 300 bp downstream of *LNCRMP (*AC092723.1). Through mechanistic studies they showed that this variant impacted IRF8 and *LNCRMP* expression through regulation of binding of the transcription factor PU.1. They went on to show that *LNCRMP* recruits TET1 to the IRF8 promoter and affects expression levels through DNA methylation. Our data support *LNCRMP* functioning as a growth regulator of monocytes most likely through its *cis* regulation of IRF8. Previous work on conditional knockdown of IRF8 in BMDMs resulted in upregulation of many genes, again supporting the idea that most of the effects of knocking down *LNCRMP* are through cis regulation of IRF8 (30). The effect we observed in the *LNCRMP* knockdown cells on IRF8 was modest. However, we had difficulty obtaining cell populations containing over 60% knockdown of *LNCRMP* (Fig. 2), perhaps because of the lethal effects of knocking this gene down resulting in loss of these cells from our population. It is also possible that there is a different start site for this gene in monocytes than the one annotated in gencode. If this is the case the targeting of our sgRNAs might not be optimal and impact on the levels of knockdown obtained. Indeed, examination of our previously generated data from primary human monocytes indicated low expression of *LNCRMP* at baseline and there are peaks of open chromatin throughout the gene body with little peaks surrounding the annotated start site (Fig. S4) (31, 32). Data from our long read isoform atlas of activated macrophages (IAMA) indicates multiple possible isoforms with highest expression in primary monocyte derived macrophages following LPS and PolyI:C stimulation (Fig. S5) (33). In THP1 cells the first exon extends beyond the annotated start site which could therefore impact the level of knockdown we are obtaining (Fig. S4). Given that *LNCRMP* is expressed in both the cytosol and the nucleus, it is possible that it has effects *in trans* outside of its role in regulating IRF8 *in cis*. Further work will be needed to fully understand the complexities of the locus and the role that *LNCRMP* plays in regulating IRF8 in myeloid cells. This work could be important as it relates to the role that these genes play in regulating disease pathogenesis in conditions such as Lupus.

*OLMALINC* appears to be more widely expressed across cell types but has only been reported to play a role in regulating cell growth in the brain and now in monocytic cells (20). We find *OLMALINC* to be strongly expressed in THP1 monocytic cells (Fig. S6). There is a strong open chromatin peak for *OLMALINC* in primary monocytes, but its expression is low by RNA-seq (Fig. S4). It is higher in expression in primary monocyte-derived macrophages, but its expression does not change following inflammatory activation (Fig. S7)*. OLMALINC* could impact different signaling pathways in various cell types, possibly through its locus being multifunctional. We find it dominantly expressed in the cytosol of monocytes and have identified putative small open reading frames that can be translated to produce a short, encoded peptide (SEP). A previous study also found *OLMALINC* to be a growth regulator in HELA cells (24), and they tiled the length of the gene using active Cas9 and found the regions containing ORF2 affected the growth phenotype, again providing more evidence for a functional SEP within the *OLMALINC* locus. In the THP1 Riboseq data we analyzed, a more upstream ORF shows strong evidence of translation, implicating *OLMALINC* as a bicistronic RNA (Fig. 3*I*). It is possible that its RNA plays roles in regulating its neighboring gene, SCD, *in cis* as previously reported (20). It is also possible that it functions to regulate SCD and other genes through its SEP, thereby impacting monocytic cell growth. Again, further mechanistic work is required to fully understand the regulatory functions of this locus across cell types.

A recent study used RNA-targeting Cas13 to pinpoint long non-coding RNAs (lncRNAs) crucial for cell survival and growth (34). They systematically targeted lncRNAs in five cell lines, including THP1 cells, and found 778 essential lncRNAs. One key finding was that most of these lncRNAs function independently of nearby protein-coding genes. This highlights a key difference between DNA-targeting Cas9 and RNA-targeting Cas13: Cas13 allows researchers to study lncRNAs that act *in trans* while we observed that CRISPRi targeting dominated for antisense transcripts and *cis-regulatory* lncRNAs. *LNCRMP* was not included in their lncRNA analysis annotation pipeline; however, they did identify *OLMALINC* (annotated as Hum_XLOC_005888) as a viability hit in THP1 cells. They also showed it was a significant hit in Hap cells, k562, and MDS-MB-231s, but not in 293 cells. Interestingly, they also noted that knocking down many of these crucial lncRNAs resulted in increased expression of interferon genes similar to what we observed for both *LNCRMP* and *OLMALINC* (34). Further work will be needed to determine if these genes are directly involved in regulating IFN pathways or if activation of these pathways is a consequence of cell death caused by knocking down genes that are essential for cell growth and proliferation.

This work demonstrates the power of CRISPR screening for rapidly identifying functional lncRNAs, a crucial step towards understanding this largely uncharacterized class of genes. Our findings, along with other recent studies, highlight the importance of *cis*-regulation by lncRNAs in fine-tuning complex signaling pathways. Notably, a recent study identified the first case of a neurological genetic disease caused by the loss of a single copy of the lncRNA CHASERR (35). This haploinsufficiency resulted in significant upregulation of a neighboring protein-coding gene, leading to disease. It is likely that many lncRNAs play similar roles in fine-tuning the expression of neighboring genes, with potential implications for understanding the pathogenesis of complex inflammatory diseases. Further investigation of these multifunctional lncRNA loci is critical for unlocking new therapeutic targets and advancing our understanding of human health.

## Methods

### Cell lines

Wildtype (WT) THP1 cells were obtained from ATCC. All THP1 cell lines were cultured in RPMI 1640 supplemented with 10% low-endotoxin fetal bovine serum (ThermoFisher), 1X penicillin/streptomycin, and incubated at 37 °C in 5% CO2.

### Lentivirus production

All constructs were cotransfected into HEK293T cells with lentiviral packaging vectors psPAX (Addgene cat#12260) and pMD2.g (Addgene cat#12259) using Lipofectamine 3000 (ThermoFisher cat# L3000001) according to the manufacturer's protocol. Viral supernatant was harvested 72 h post-transfection.

### Screening protocol

#### sgRNA library design and cloning

10 sgRNAs were designed for each TSS of hg19 annotated lncRNAs expressed in THP1s at baseline and upon stimulation. The sgRNA library also included 700 non-targeting control sgRNAs, and sgRNAs targeting 50 protein-coding genes as positive controls. The sgRNA library was designed and cloned as previously described in (16).

#### Screening and analysis

THP1-CRISPRi cells were infected with the sgRNA genome-scale library at a at low multiplicity of infection (MOI = 0.3). Three days post infection, cells were puromycin-selected (10 μg/ml) for 5 days to obtain cherry-positive (sgRNA) cells and were maintained at >1000X coverage at all times. Cells were grown for a total of 21 days and maintained at over 1000X coverage throughout. Genomic DNA was extracted from triplicate day 21 samples and libraries prepared and sequenced as previously described (16). For the growth screen, the Day 21 sample was compared to the plasmid library pool. fastq.gz files were analyzed using the gRNA_tool: https://github.com/quasiben/gRNA_Tool. All guide RNA (sgRNA) were collapsed to obtain raw sgRNA counts. Counts were normalized to the median and fold-changes were calculated for each sgRNA. To identify significant genes for the growth screen, the Mann-Whitney U test was performed comparing fold-changes for sgRNAs targeting each gene to non-targeting controls (described in (36)). Data is available at GSE278486.

### Sequencing data

RNA sequencing was performed to compare negative control THP1 cells to those with *OLMALINC* (labeled KO 823) or *LNCRMP* (labeled KO 667) knockdown. Data are available at GSE278485.

### THP1-dCasKRAB-sgRNA

CRISPRi-THP1 cells were lentivirally infected with sgRNAs. sgRNA constructs were made from a pSico lentiviral backbone driven by an EF1a promoter expressing T2A flanked genes: puromycin resistance and mCherry. sgRNAs were expressed from a mouse U6 promoter. 20-Nucleotide forward/reverse gRNA oligonucleotides were annealed and cloned *via* the AarI site.

**Table:** Control and target sgRNAs

Gene name	sgRNA sequence
ENSG00000269667_g1	GGGGATAAGCAGCCAAGGGG
ENSG00000269667_g2	GGGAAGTCCTGGGCTGCGCA
ENSG00000269667_g3	GGCACACTAGTGCAGCAAC
ENSG00000235823_g1	GTCCATAGCTGCCTTTGAGG
ENSG00000235823_g2	GTCTGCCCTACCCTGGAATG
ENSG00000235823_g3	GCCGCATTTTCCCCATTCCA
CASP8AP2-g1F	GCGGTGGGCAGAAAGGAACC
CASP8AP2-g2F	GATGCCAGGGAGACCTCGGT
CASP8AP2-g3F	GGAGACCTCGGTGGGCAGAA
Neg_Ctl_g1	CTGTGCAATCCGCATGATAT
Neg_Ctl_g2	ATCTGGCACCTCACCCACGT
Neg_Ctl_g3	TCATCTTCAACCGAGTTTGA

### Mixed cell growth assay

We used the top three performing sgrna guides from the screen to validate the lncrna genes. Briefly, sgRNA-infected THP1 cells (cherry-pos) were mixed with uninfected THP1 cells (cherry-neg) at a 1:1 ratio in triplicate. We used Flow cytometry to monitor the ratio of cherry-pos to cherry-neg cells at 0- and 21-days post-plating. All validation cytometry was performed on the Attune NxT Flow Cytometer.

### Nuc/Cyt fractionation and RT-qPCR

WT THP1 cells were fractionated according to the NE-PER kit protocol (ThermoFisher cat# 78833) with RNAse inhibitor (Superase-IN, ThermoFisher cat# AM2696) added to the cytosolic and nuclear lysis buffers. 3 volumes of Trizol (TRI Reagent, Sigma T9424) were added to the fractions, and RNA was isolated using the DIrect-zol RNA Miniprep Plus Kit (Zymo cat# R2052). 16uL of RNA isolated from fractions was reverse transcribed (iScript cDNA synthesis kit, Bio-Rad cat# 1708840) followed by qPCR (iTaq SYBRgreen Supermix, Bio-Rad cat# 1725121) using the cycling conditions as follows: 50C for 2 min, 95 °C for 2 min followed by 40 cycles of 95 °C for 15s, 60 °C for 30s, and 72 °C for 45s.

Primers:

**Table:** 

ENSG00000269667	
*LNCRMP*F1	GATGGATCCGACTTGTGGTT
*LNCRMP*R1	AAAAGAAATGCGCTCAAGGA
ENSG00000235823	
*OLMALINC*_F	AGTCTGCCCTACCCTGGAAT
*OLMALINC*_R	ATTGGCAAGATGTTCCTTGG
huHPRT	
ATCAGACTGAAGAGCTATTGT	TGGCTTATATCCAACACTTCG

RT-qPCR analysis was performed using GraphPad Prism. Statistical significance was determined using a *t* test and a *p-value* of less than 0.05 was considered significant.

## Data availability

All screening data can be found at GSE278486. All RNA-sequencing data can be found at GSE278485.

## Supporting information

This article contains supporting information.

## Conflict of interest

The authors declare the following financial interests/personal relationships which may be considered as potential competing interests: S.C. is a paid consultant for NextRNA therapeutics.

## References

[1] Kleiveland C.R., Verhoeckx K., Cotter P., López-Expósito I., Kleiveland C., Lea T., Mackie A. (2015). The Impact of Food Bioactives on Health: In Vitro and Ex Vivo Models.

[2] Shi C., Pamer E.G. (2011). Monocyte recruitment during infection and inflammation. Nat. Rev. Immunol..

[3] Auffray C., Sieweke M.H., Geissmann F. (2009). Blood monocytes: development, heterogeneity, and relationship with dendritic cells. Annu. Rev. Immunol..

[4] Parihar A., Eubank T.D., Doseff A.I. (2010). Monocytes and macrophages regulate immunity through dynamic networks of survival and cell death. J. Innate Immun..

[5] Karlmark K.R., Tacke F., Dunay I.R. (2012). Monocytes in health and disease – minireview. Eur. J. Microbiol. Immunol. (Bp).

[6] Pang J., Maienschein-Cline M., Koh T.J. (2022). Monocyte/macrophage heterogeneity during skin wound healing in mice. J. Immunol..

[7] Mattick J.S., Amaral P.P., Carninci P., Carpenter S., Chang H.Y., Chen L.-L. (2023). Long non-coding RNAs: definitions, functions, challenges and recommendations. Nat. Rev. Mol. Cell Biol..

[8] Flynn R.A., Chang H.Y. (2014). Long noncoding RNAs in cell-fate programming and reprogramming. Cell Stem Cell.

[9] Montano C., Covarrubias S., Malekos E., Katzman S., Carpenter S. (2024). Identification and functional characterization of lncRNAs involved in human monocyte-to-macrophage differentiation. bioRxiv.

[10] Robinson E.K., Covarrubias S., Carpenter S. (2020). The how and why of lncRNA function: an innate immune perspective. Biochim. Biophys. Acta Gene Regul. Mech..

[11] Huarte M. (2015). The emerging role of lncRNAs in cancer. Nat. Med..

[12] Mirsafian H., Manda S.S., Mitchell C.J., Sreenivasamurthy S., Ripen A.M., Mohamad S.B. (2016). Long non-coding RNA expression in primary human monocytes. Genomics.

[13] Malmström E., Khan H.N., Veer C.V., Stunnenberg M., Meijer M.T., Matsumoto H. (2022). The long non-coding antisense RNA JHDM1D-AS1 regulates inflammatory responses in human monocytes. Front. Cell. Infect. Microbiol..

[14] Yang C.-A., Li J.-P., Yen J.-C., Lai I.-L., Ho Y.-C., Chen Y.-C. (2018). lncRNA NTT/PBOV1 Axis promotes monocyte differentiation and is elevated in rheumatoid arthritis. Int. J. Mol. Sci..

[15] Liu S.J., Horlbeck M.A., Cho S.W., Birk H.S., Malatesta M., He D. (2017). CRISPRi-based genome-scale identification of functional long non-coding RNA loci in human cells. Science.

[16] Halasz H., Malekos E., Covarrubias S., Yitiz S., Montano C., Sudek L. (2024). CRISPRi screens identify the lncRNA, LOUP, as a multifunctional locus regulating macrophage differentiation and inflammatory signaling. Proc. Natl. Acad. Sci. U. S. A..

[17] Cho S.W., Xu J., Sun R., Mumbach M.R., Carter A.C., Chen Y.G. (2018). Promoter of lncRNA gene PVT1 is a tumor-suppressor DNA boundary element. Cell.

[18] Myacheva K., Walsh A., Riester M., Pelos G., Carl J., Diederichs S. (2023). CRISPRi screening identifies CASP8AP2 as an essential viability factor in lung cancer controlling tumor cell death via the AP-1 pathway. Cancer Lett..

[19] Hummon A.B., Pitt J.J., Camps J., Emons G., Skube S.B., Huppi K. (2012). Systems-wide RNAi analysis of CASP8AP2/FLASH shows transcriptional deregulation of the replication-dependent histone genes and extensive effects on the transcriptome of colorectal cancer cells. Mol. Cancer.

[20] Mills J.D., Kavanagh T., Kim W.S., Chen B.J., Waters P.D., Halliday G.M. (2015). High expression of long intervening non-coding RNA OLMALINC in the human cortical white matter is associated with regulation of oligodendrocyte maturation. Mol. Brain.

[21] Covarrubias S., Vollmers A.C., Capili A., Boettcher M., Shulkin A., Correa M.R. (2020). High-throughput CRISPR screening identifies genes involved in macrophage viability and inflammatory pathways. Cell Rep..

[22] Benhammou J.N., Ko A., Alvarez M., Kaikkonen M.U., Rankin C., Garske K.M. (2019). Novel lipid long intervening noncoding RNA, oligodendrocyte maturation-associated long intergenic noncoding RNA, regulates the liver steatosis gene stearoyl-coenzyme A desaturase as an enhancer RNA. Hepatol. Commun..

[23] Hah N., Benner C., Chong L.W., RT Y.U., Downes M. (2015). Inflammation-sensitive super enhancers form domains of coordinately regulated enhancer RNAs. Proc. Natl. Acad. Sci. U.S.A..

[24] Prensner J.R., Enache O.M., Luria V., Krug K., Clauser K.R., Dempster J.M. (2021). Noncanonical open reading frames encode functional proteins essential for cancer cell survival. Nat. Biotechnol..

[25] Zhou T., Zhu X., Ye Z., Wang Y.-F., Yao C., Xu N. (2022). Lupus enhancer risk variant causes dysregulation of IRF8 through cooperative lncRNA and DNA methylation machinery. Nat. Commun..

[26] Langlais D., Barreiro L.B., Gros P. (2016). The macrophage IRF8/IRF1 regulome is required for protection against infections and is associated with chronic inflammation. J. Exp. Med..

[27] Yáñez A., Ng M.Y., Hassanzadeh-Kiabi N., Goodridge H.S. (2015). IRF8 acts in lineage-committed rather than oligopotent progenitors to control neutrophil vs monocyte production. Blood.

[28] Kurotaki D., Osato N., Nishiyama A., Yamamoto M., Ban T., Sato H. (2013). Essential role of the IRF8-KLF4 transcription factor cascade in murine monocyte differentiation. Blood.

[29] Gaillard C., Surianarayanan S., Bentley T., Warr M.R., Fitch B., Geng H. (2018). Identification of IRF8 as a potent tumor suppressor in murine acute promyelocytic leukemia. Blood Adv..

[30] Twum D.Y., Colligan S.H., Hoffend N.C., Katsuta E., Cortes Gomez E., Hensen M.L. (2019). IFN regulatory factor-8 expression in macrophages governs an antimetastatic program. JCI Insight.

[31] Robinson E.K., Jagannatha P., Covarrubias S., Cattle M., Smaliy V., Safavi R. (2021). Inflammation drives alternative first exon usage to regulate immune genes including a novel iron-regulated isoform of Aim2. Elife.

[32] Vollmers A.C., Mekonen H.E., Campos S., Carpenter S., Vollmers C. (2021). Generation of an isoform-level transcriptome atlas of macrophage activation. J. Biol. Chem..

[33] Song R., Gao Y., Dozmorov I., Malladi V., Saha I., McDaniel M.M. (2021). IRF1 governs the differential interferon-stimulated gene responses in human monocytes and macrophages by regulating chromatin accessibility. Cell Rep..

[34] Liang W.-W., Müller S., Hart S.K., Wessels H.-H., Méndez-Mancilla A., Sookdeo A. (2024). Transcriptome-scale RNA-targeting CRISPR screens reveal essential lncRNAs in human cells. Cell.

[35] Ganesh V.S., Riquin K., Chatron N., Yoon E., Lamar K.-M., Aziz M.C. (2024). Neurodevelopmental disorder caused by deletion of CHASERR, a lncRNA gene. N. Engl. J. Med..

[36] Gilbert L.A., Horlbeck M.A., Adamson B., Villalta J.E., Chen Y., Whitehead E.H. (2014). Genome-scale CRISPR-mediated control of gene repression and activation. Cell.

