# Supplementary material for: CRISPRi screen uncovers lncRNA regulators of human monocyte growth
Source: J Biol Chem. 2025 May 7;301(6):110204. doi: 10.1016/j.jbc.2025.110204 (PMC12167476; doi:10.1016/j.jbc.2025.110204)
Supplement: Supplementary Fig 1 [file mmc1.pdf]

Supp.Fig.1

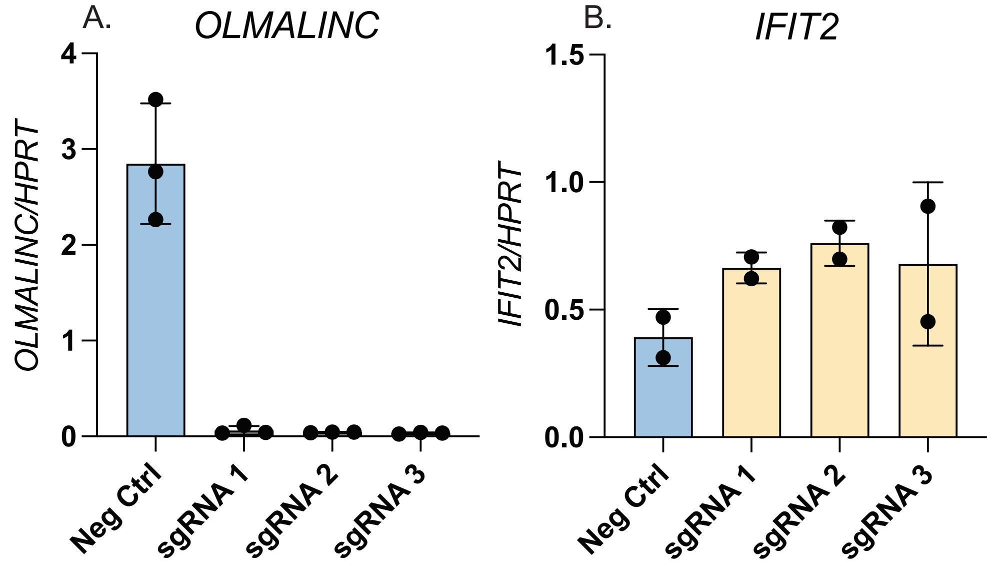

Supplemental Figure 1: Knockdown of *OLMALINC* results in increased expression of *IFIT2*. A-B. THP1 cells were infected with a control sgRNA (Neg Ctl) or 3 sgRNAs targeting *OLMALINC*. Expression of *OLMALINC* or *IFIT2* was measured by qPCR. Error bars indicate standard deviation of biological triplicates for *OLMALINC* and biological duplicates for *IFIT2*.
