# Supplementary material for: CRISPRi screen uncovers lncRNA regulators of human monocyte growth
Source: J Biol Chem. 2025 May 7;301(6):110204. doi: 10.1016/j.jbc.2025.110204 (PMC12167476; doi:10.1016/j.jbc.2025.110204)
Supplement: Supplementary Fig 2 [file mmc2.pdf]

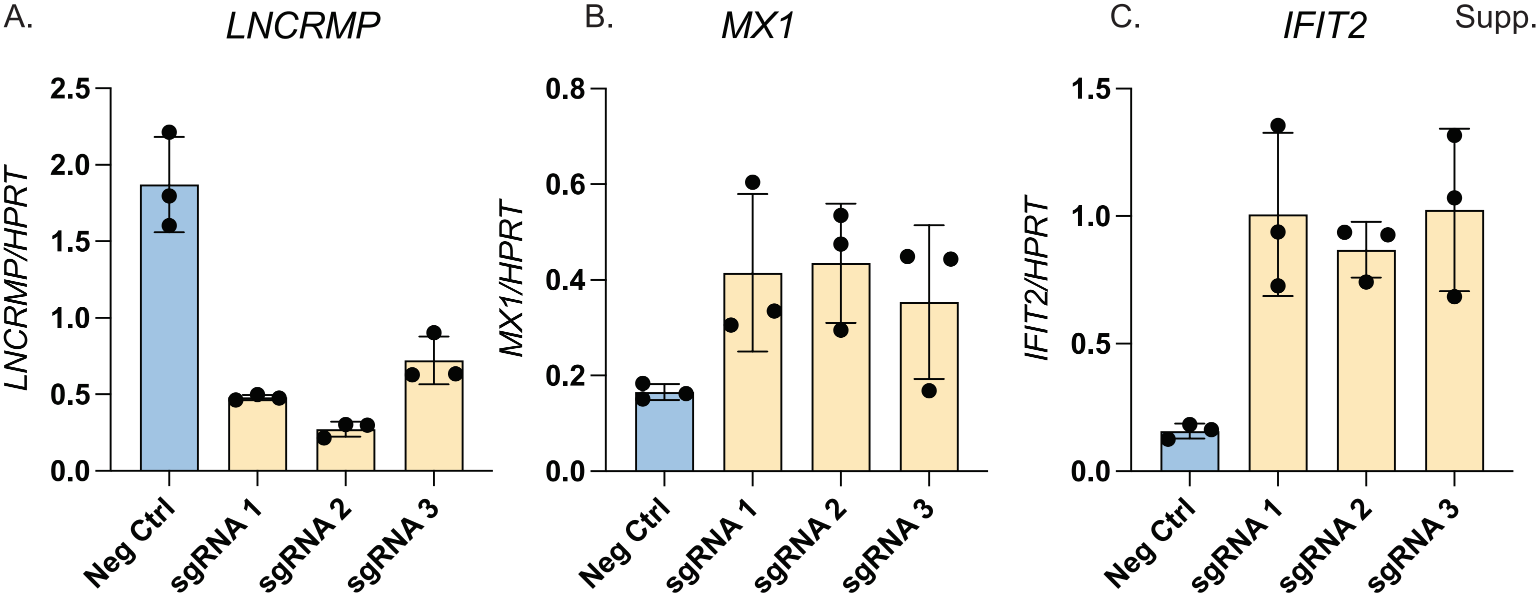

Supplemental Figure 2: Knockdown of *LNCRMP* results in increased expression of *MX1* and *IFIT2*. A-C. THP1 cells were infected with a control sgRNA (Neg Ctl) or 3 sgRNAs targeting *LNCRMP*. Expression of *LNCRMP (A), MX1 (B) and IFIT2 (C)* was measured by qPCR. Error bars indiciate standard deviation of biological triplicates.
