# Supplementary material for: CRISPRi screen uncovers lncRNA regulators of human monocyte growth
Source: J Biol Chem. 2025 May 7;301(6):110204. doi: 10.1016/j.jbc.2025.110204 (PMC12167476; doi:10.1016/j.jbc.2025.110204)
Supplement: Supplementary Fig 3 [file mmc3.pdf]

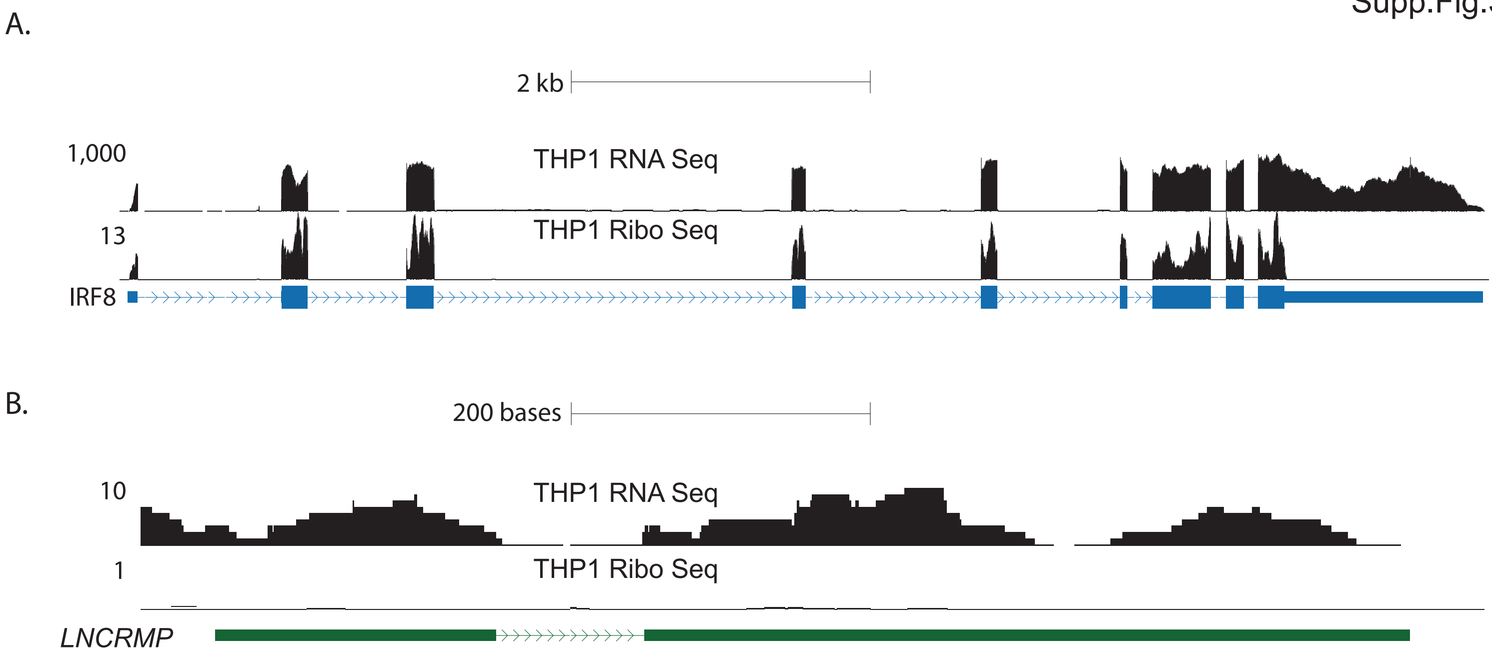

Supplemental Figure 3: ***LNCRMP*** **does not encode any short peptides.** (A,B) RNASeq and Riboseq data for *IRF8* and *LNCRMP*. *LNCRMP* does not show Riboseq aggregates indicating there is no translation of this gene.
