# Supplementary material for: CRISPRi screen uncovers lncRNA regulators of human monocyte growth
Source: J Biol Chem. 2025 May 7;301(6):110204. doi: 10.1016/j.jbc.2025.110204 (PMC12167476; doi:10.1016/j.jbc.2025.110204)
Supplement: Supplementary Fig 4 [file mmc4.pdf]

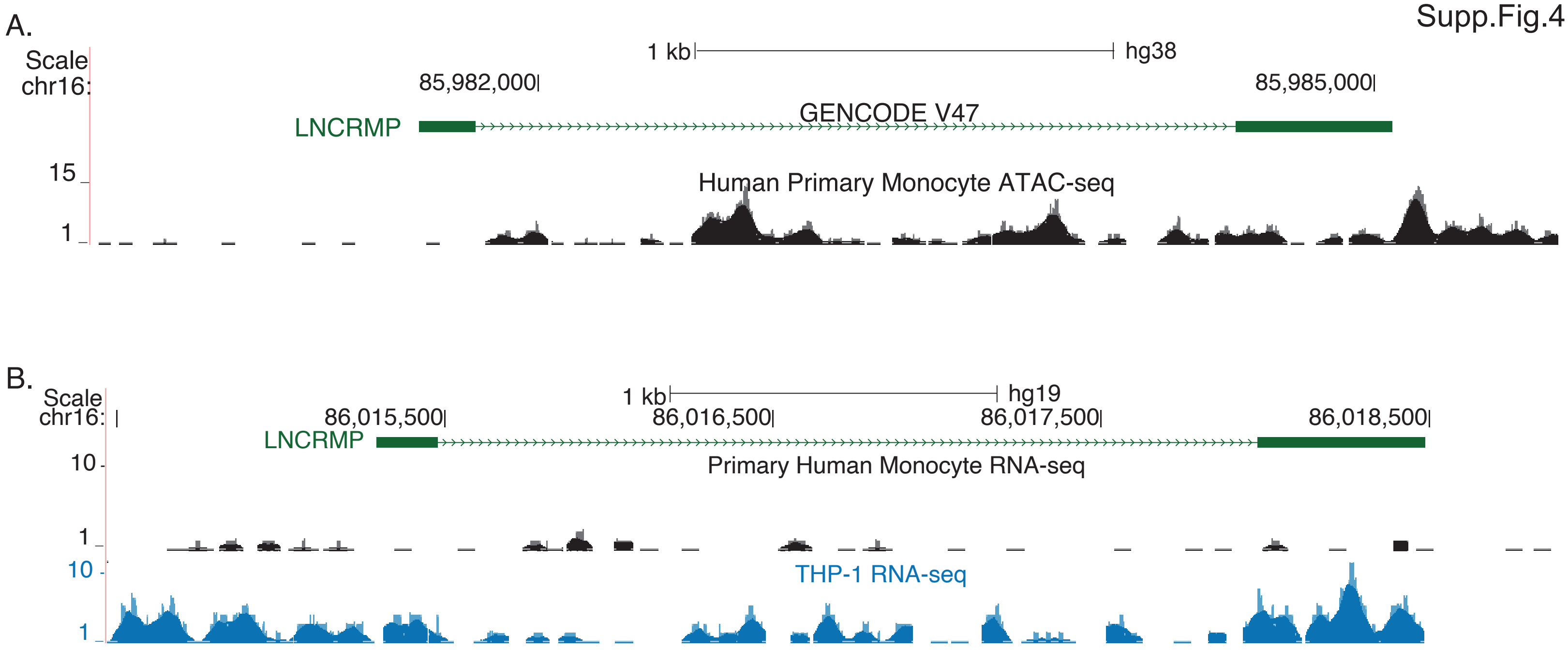

Supplemental Figure 4: *LNCRMP* locus in primary monocytes and THP1 cells. A. UCSC browser track displaying ATAC-seq data from primary monocyte at the *LNCRMP* locus. B. UCSC browser track of RNA-seq data from primary human monocytes and THP1 cells at the *LNCRMP* locus.
