# Supplementary material for: CRISPRi screen uncovers lncRNA regulators of human monocyte growth
Source: J Biol Chem. 2025 May 7;301(6):110204. doi: 10.1016/j.jbc.2025.110204 (PMC12167476; doi:10.1016/j.jbc.2025.110204)
Supplement: Supplementary Fig 5 [file mmc5.pdf]

Supp.Fig.5

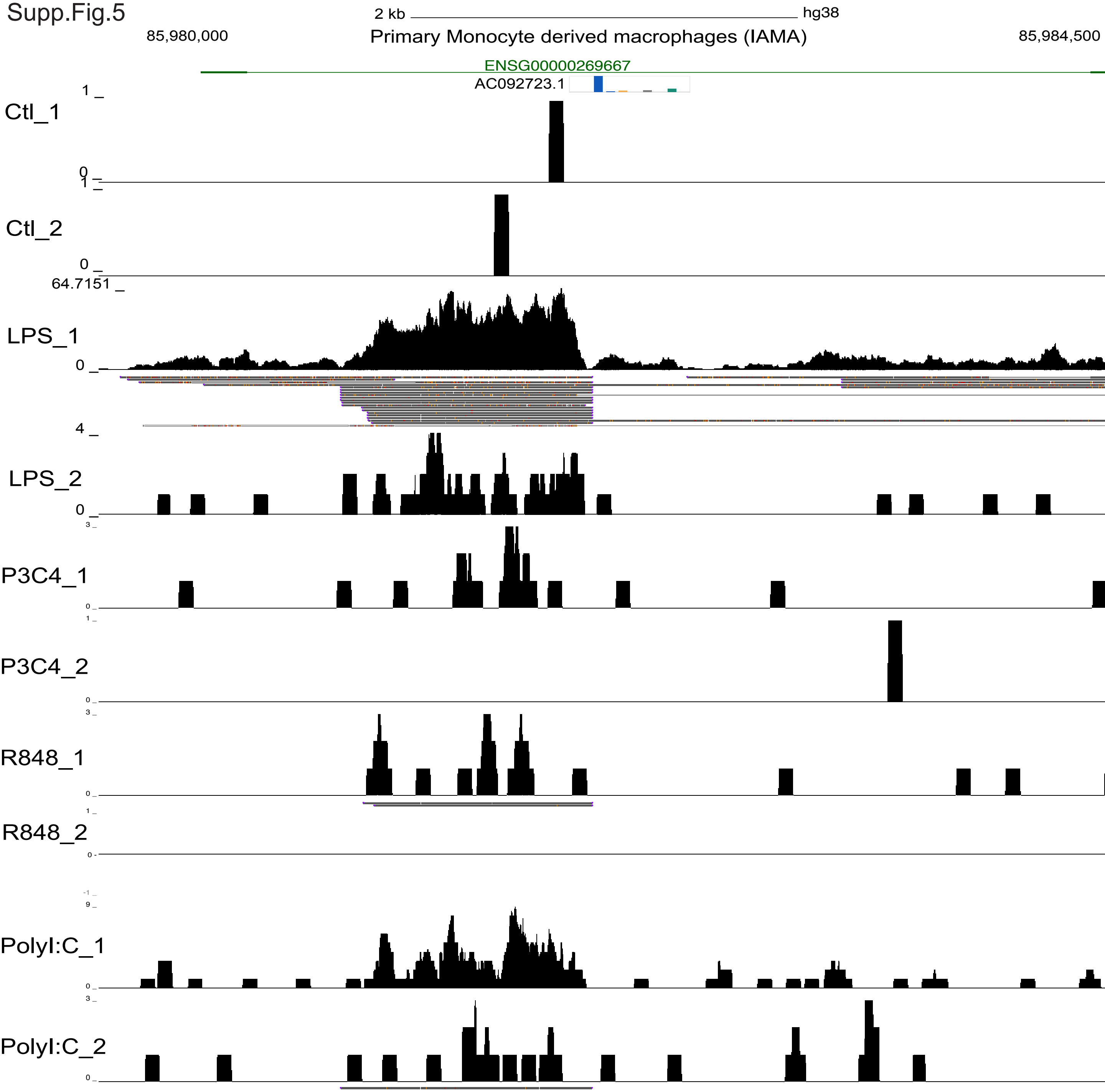

Supplemental Figure 5: *LNCRMP* locus in primary monocytes derived macrophages. UCSC browser track displaying RNA-seq from primary monocyte derived macrophages at baseline (Ctl) or following stimulation with various TLR ligands at the *LNCRMP* locus.
