# Supplementary material for: CRISPRi screen uncovers lncRNA regulators of human monocyte growth
Source: J Biol Chem. 2025 May 7;301(6):110204. doi: 10.1016/j.jbc.2025.110204 (PMC12167476; doi:10.1016/j.jbc.2025.110204)
Supplement: Supplementary Fig 6 [file mmc6.pdf]

Supp.Fig.6

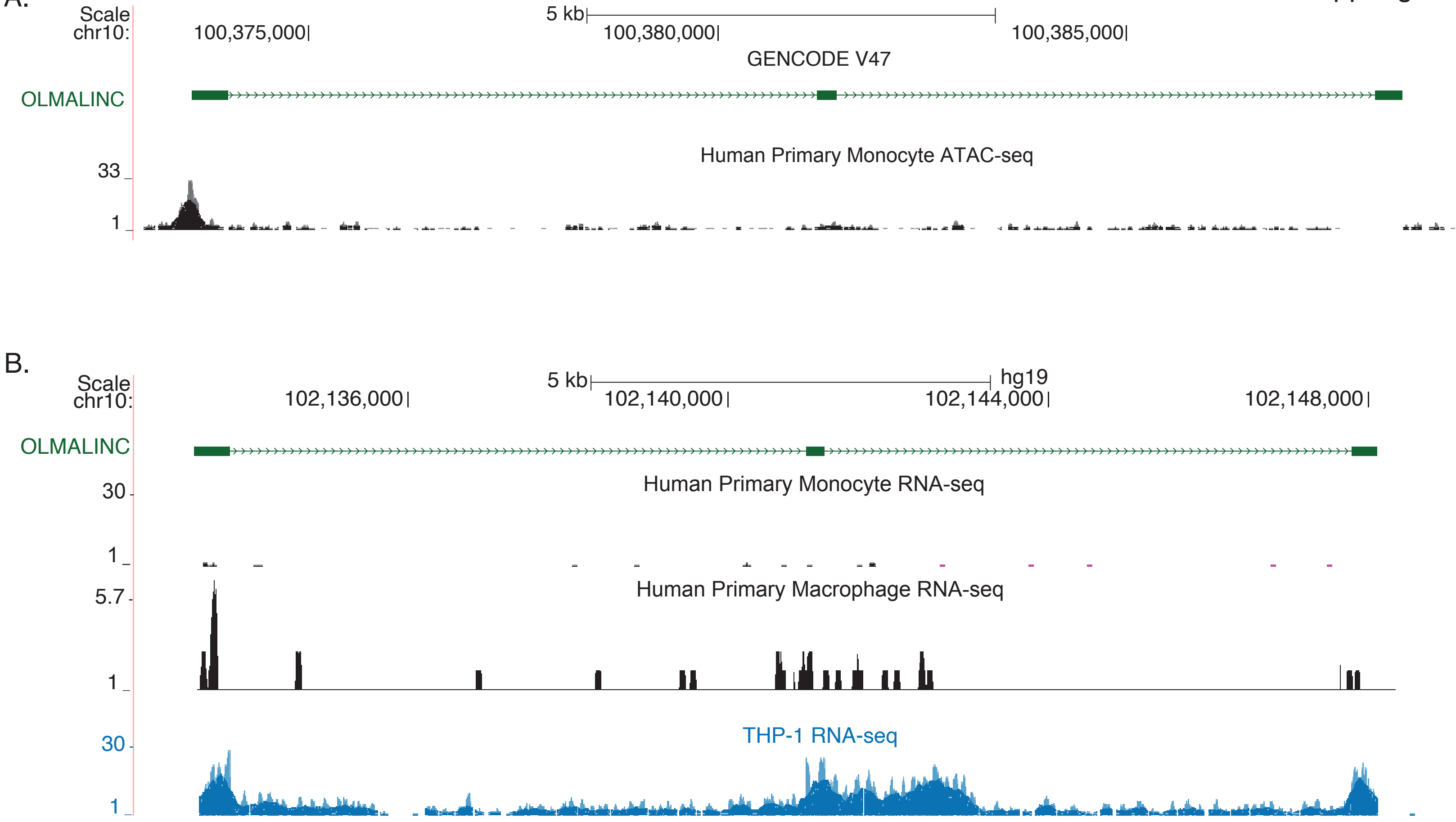

Supplemental Figure 6: *OLMALINC* locus in primary monocytes, macrophages and THP1 cells.
A. UCSC browser track displaying ATAC-seq data from primary monocyte at the *OLMALINC* locus.
B. UCSC browser track of RNA-seq data from primary human monocytes, macrophages and THP1 cells at the *OLMALINC* locus.
