# Supplementary material for: CRISPRi screen uncovers lncRNA regulators of human monocyte growth
Source: J Biol Chem. 2025 May 7;301(6):110204. doi: 10.1016/j.jbc.2025.110204 (PMC12167476; doi:10.1016/j.jbc.2025.110204)
Supplement: Supplementary Table 1 [file mmc8.docx]

Table S1


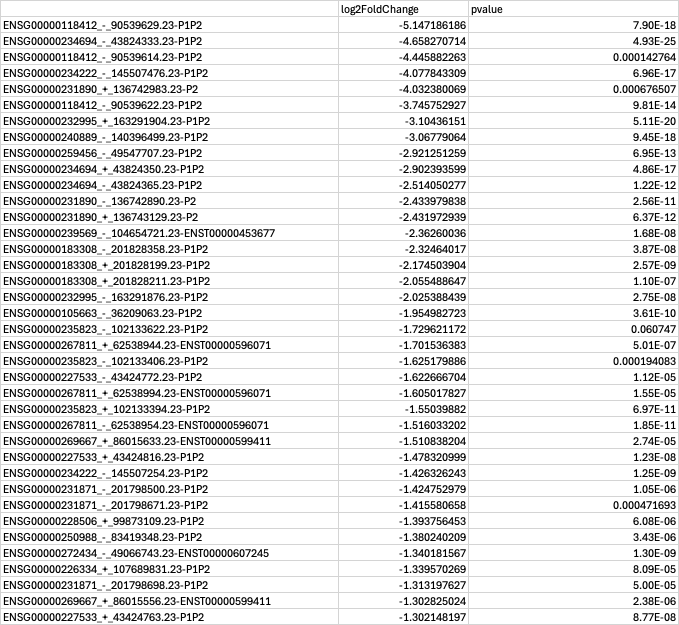


**Table S1: Positive regulator of THP1 growth from CRISPRi screen.**

guide RNA (sgRNA) were collapsed to obtain raw sgRNA counts. Counts were normalized to the median and fold-changes were calculated for each sgRNA.

Table S2

| **Gene ID** | **LncRNA Gene Name** | **Viability lncrna identified as viability gene GenomeCRISPR screens** | **lncRNA Class and direction** | **Neighboring protein coding gene** | **Distance from viability lncrna** | **Type of regulator** | **Neighboring gene identified as viability gene on GenomeCRISPR screens** |
| --- | --- | --- | --- | --- | --- | --- | --- |
| ENSG00000269667 | LNCRMP | No information  available | Intergenic lncRNA: forward strand | IRF8 (downstream) | -84433 | cis regulator | Yes (including THP1) |
|  |  |  |  | FOXF1 (upstream) | 525646 | cis regulator | Yes (does not include THP1) |
| ENSG00000235823 | OLMALINC | No information  available | Intergenic lncRNA: forward strand | WNT8B | -84550 |  | No hits on viability screens |
|  |  |  |  | SCD | 31367 | cis regulator | Yes (including THP1) |
| ENSG00000228506 | RP11-98I9.4 | no | Intergenic lncRNA: forward strand | PNISR | -2839 | cis regulator | Yes (does not include THP1) |
|  |  |  |  | USP45 | 87229 |  | No hits on viability screens |
| ENSG00000271797 | CTC-428G20.6 | no | Intergenic lncRNA: novel transcript, antisense to PGGT1B forward strand. | PGGT1B | -104 | Overlaping protein | Yes (does not include THP1) |
|  |  |  |  | CCDC112 | 3742 | cis regulator | No hits on viability screens |
| ENSG00000232388 | SMIM26 | No information  available | Protein coding | DTD1 | -19402 | cis regulator | No hits on viability screens |
|  |  |  |  | SEC23B | 60936 | cis regulator | Yes (including THP1) |
| ENSG00000255857 | PXN-AS1 | No information  available | Intergenic lncRNA: PXN antisense RNA 1, antisense  forward strand | RPLP0 | -6115 | cis regulator | Yes (including THP1) |
|  |  |  |  | PXN | 58546 | cis regulator | Yes (does not include THP1) |
| ENSG00000231890 | DARS1-AS1 | Yes | Intergenic lncRNA: DARS1 antisense RNA 1 forward strand | DARS | -18063 | cis regulator | Yes (including THP1) |
|  |  |  |  | CXCR4 | 91756 | cis regulator | No hits on viability screens |
| ENSG00000223473 | GS1-124K5.3 | No information  available | Intergenic lncRNA: | TPST1 | 285851 |  | No hits on viability screens |
|  |  |  |  | KCTD7 | 149513 |  | No hits on viability screens |
| ENSG00000244625 | MIATNB | No information  available | Intergenic lncRNA: Forward strand | HPS4 | 221311 |  | No hits on viability screens |
|  |  |  |  | CRYBA4 | 50827 |  | No hits on viability screens |
| ENSG00000267811 | RP11-727F15.11 | No information  available | Antisense lncRNA: (-): antisense to TAF6L reverse strand | TMEM179B | -16064 | cis regulator | Yes (does not include THP1) |
|  |  |  |  | TAF6L | 60 | overlapping protein | Yes (does not include THP1) |
| ENSG00000254027 | RP11-363E6.3 | No information  available | Antisense lncRNA: novel transcript reverse strand | FABP5 | 84433 | cis regulator | Yes (does not include THP1) |
|  |  |  |  |  |  |  |  |
| ENSG00000231871 | IPO9-AS1 | Yes (1 Screen) | Antisense lncRNA: IPO9 antisense RNA 1 reverse strand | IPO9 | -70232 | cis regulator | Yes (does not include THP1) |
|  |  |  |  | NAV1 | 0 | overlapping protein | Yes (does not include THP1) |
| ENSG00000239569 | KMT2E-AS1 | No information  available | Antisense lncRNA: KMT2E antisense RNA 1 (head to head), reverse strand | KMT2E | 0 | overlapping protein | Yes (does not include THP1) |
|  |  |  |  |  |  |  |  |
| ENSG00000240889 | NDUFB2-AS1 | Yes (multiple screens) | Antisense lncRNA: NDUFB2 antisense RNA 1 reverse strand | NDUFB2 | 0 | overlapping protein | Yes (does not include THP1) |
|  |  |  |  | ADCK2 | 0 | overlapping protein | No hits on viability screens |
| ENSG00000271918 | CTD-2287O16.5 | No information  available | Antisense lncRNA: antisense reverse strand | COMMD10 | -380 | overlapping protein | Yes (does not include THP1) |
|  |  |  |  |  |  |  |  |
| ENSG00000230910 | ADGRB3-DT | No information  available | Bidirectional lncRNA: antisense reverse strand | BAI3 |  |  | No hits on viability screens |
| ENSG00000234694 | CDC20-DT | No information  available | Bidirectional lncRNA: | CDC20 | -2342 |  | Yes (including THP1) |
|  |  |  |  | MPL | +18864 |  | Yes (does not include THP1) |
| ENSG00000272711 | HK2-DT | No information  available | Antisense lncRNA: HK2 divergent transcript reverse strand | HK2 | -660 | overlapping protein | Yes (does not include THP1) |
|  |  |  |  |  |  |  |  |
| ENSG00000241764 | AC002467.7 | No information  available | Antisense lncRNA: antisense reverse strand | CBLL1 | 2 | overlapping protein | Yes (does not include THP1) |
|  |  |  |  | SLC26A3 | 59508 | cis regulator | No hits on viability screens |
| ENSG00000259456 | ADNP-AS1 | No information  available | Antisense lncRNA: ADNP antisense lncRNA (+) forward strand | ADNP | -6846 | cis regulator | Yes (does not include THP1) |
|  |  |  |  | DPM1 | 20491 | cis regulator | Yes (does not include THP1) |
| ENSG00000250988 | SNHG21 | No information  available | Antisense lncRNA: small nucleolar RNA host gene 21 forward strand. | WHAMM | -84550 | cis regulator | Yes (does not include THP1) |
|  |  |  |  | AP3B2 | 31367 | cis regulator | Yes (does not include THP1) |
| ENSG00000272434 | RP13-131K19.6 | No information  available | Antisense lncRNA: novel transcript, antisense to IMPDH2 and QRICH1, forward strand | IMPDH2 | -121 | overlapping protein | Yes (does not include THP1) |
|  |  |  |  | QRICH1 | 64852 | long cis regulator | Yes (does not include THP1) |
| ENSG00000259460 | RP11-128A17.1 | No information  available | Antisense lncRNA: novel transcript, antisense to MEIS2,forward strand | MEIS2 | 129 | overlapping protein | Yes (does not include THP1) |
| ENSG00000183308 | AC005037.3 | No information  available | Antisense lncRNA: forward strand antisense | ORC2 | -22502 | cis regulator | Yes (does not include THP1) |
|  |  |  |  | FAM126B | 85487 | cis regulator | Yes (does not include THP1) |
| ENSG00000227533 | SLC2A1-AS1 | No information  available | Antisense lncRNA: SLC2A1 antisense RNA 1 forward strand | SLC2A1 | -12373 | cis regulator | Yes (does not include THP1) |
| ENSG00000234996 | ACTG1P25 | No information  available | Antisense lncRNA: RP11-480I12.7 forward strand | RABIF | 20638 | cis regulator | Yes (does not include THP1) |
| ENSG00000257303 | RP11-977G19.11 | No information  available | Antisense lncRNA: antisense forward strand | CS | -7083 | cis regulator | Yes (does not include THP1) |
|  |  |  |  | CNPY2 | 8861 | cis regulator | Yes (does not include THP1) |
| ENSG00000226334 | RP11-217B7.2 | No information  available | Antisense lncRNA: forward strand | ABCA1 | 15 | overlapping protein | No hits on viability screens |
| ENSG00000227533 | SLC2A1-AS1 | No information  available | Antisense lncRNA: SLC2A1 antisense RNA 1 forward strand | SLC2A1 | -12373 | cis regulator | Yes (does not include THP1) |
| ENSG00000267390 | KDSR-DT | No information  available | Antisense lncRNA: RP11-635N19.1 antisense forward strand | KDSR | -6968 | cis regulator | Yes (does not include THP1) |
|  |  |  |  | VPS4B | 47985 | cis regulator | Yes (does not include THP1) |
| ENSG00000234222 | RP11-315I20.1 | Yes (several viability screens) | Sense intronic lncRNA: LIX1L antisense RNA 1 forward strand | RBM8A | -15356 | cis regulator | Yes (including THP1) |
| ENSG00000262944 |  | No information  available | Sense intronic lncRNA: RP11 forward strand (cancer) | LIX1L | 15157 | cis regulator | Yes (does not include THP1) |
| ENSG00000232995 | LOC127814295 | No information  available | Sense intronic lncRNA: RP11 reverse strand | RGS5 | -80296 | cis regulator | Yes (does not include THP1) |
|  |  |  |  | NUF2 | -38755 | cis regulator | Yes (including THP1) |

| **Gene ID** | **Gene Name** | **Gene identified as viability gene GenomeCRISPR screens** | **lncRNA Class and direction** | **Neighboring protein coding gene** | **Distance from viability gene** | **Type of regulator** | **Neighboring gene identified as viability gene on GenomeCRISPR screens** |
| --- | --- | --- | --- | --- | --- | --- | --- |
| ENSG00000118412 | CASP8AP2 | yes | CASP8AP2 forward strand | GJA10 | -42304 | Unknown | No hits on viability screens |
|  |  |  |  | MDN1 | -32442 | Unknown | Yes (including THP1) |
| ENSG00000105663 | KMT2B |  |  | ENSG00000272333 | +10429 | Unknown |  |
|  |  |  |  | IGFLR1 | +13998 | Unknown |  |
| ENSG00000232388 | SMIM26 | No information  available | Protein coding | DTD1 | -19402 | cis regulator | No hits on viability screens |
|  |  |  |  | SEC23B | 60936 | cis regulator | Yes (including THP1) |
| ENSG00000135253 | KPC | Yes (multiple screens) | Sense intronic lncRNA: kielin/chordin-like protein, reverse strand (cancer) | IRF5 | -51355 | cis regulator | Yes (does not include THP1) |
|  |  |  |  | ATP6V1F | 23681 | cis regulator | Yes (includes THP1) |

**Table S2: Top hits and their neighboring genes:** Table outlines each hit with its neighboring protein coding genes and whether these proteins have emerged as hits in previous viability screens from GenomeCRISPR.
